# Supplementary figures and images for: Dissecting the Regulatory Network of Leaf Premature Senescence in Maize (Zea mays L.) Using Transcriptome Analysis of ZmELS5 Mutant
Source: Genes (Basel). 2019 Nov 19;10(11):944. doi: 10.3390/genes10110944 (PMC6895817; doi:10.3390/genes10110944)

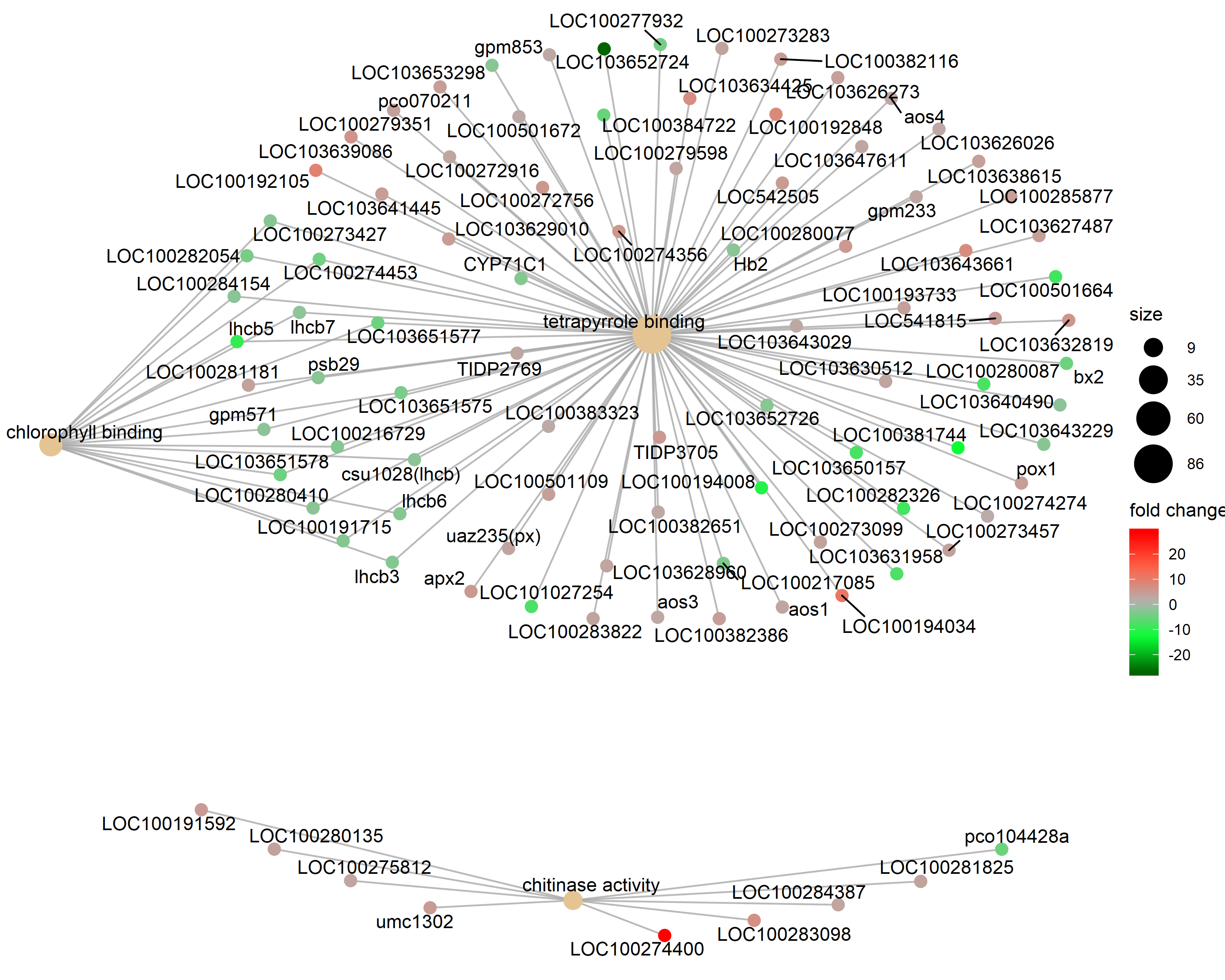

Supplement: Supplementary file 1 [file genes-10-00944-s001.zip › 补充数据/Figure S1. Different molecular function connect network.png]

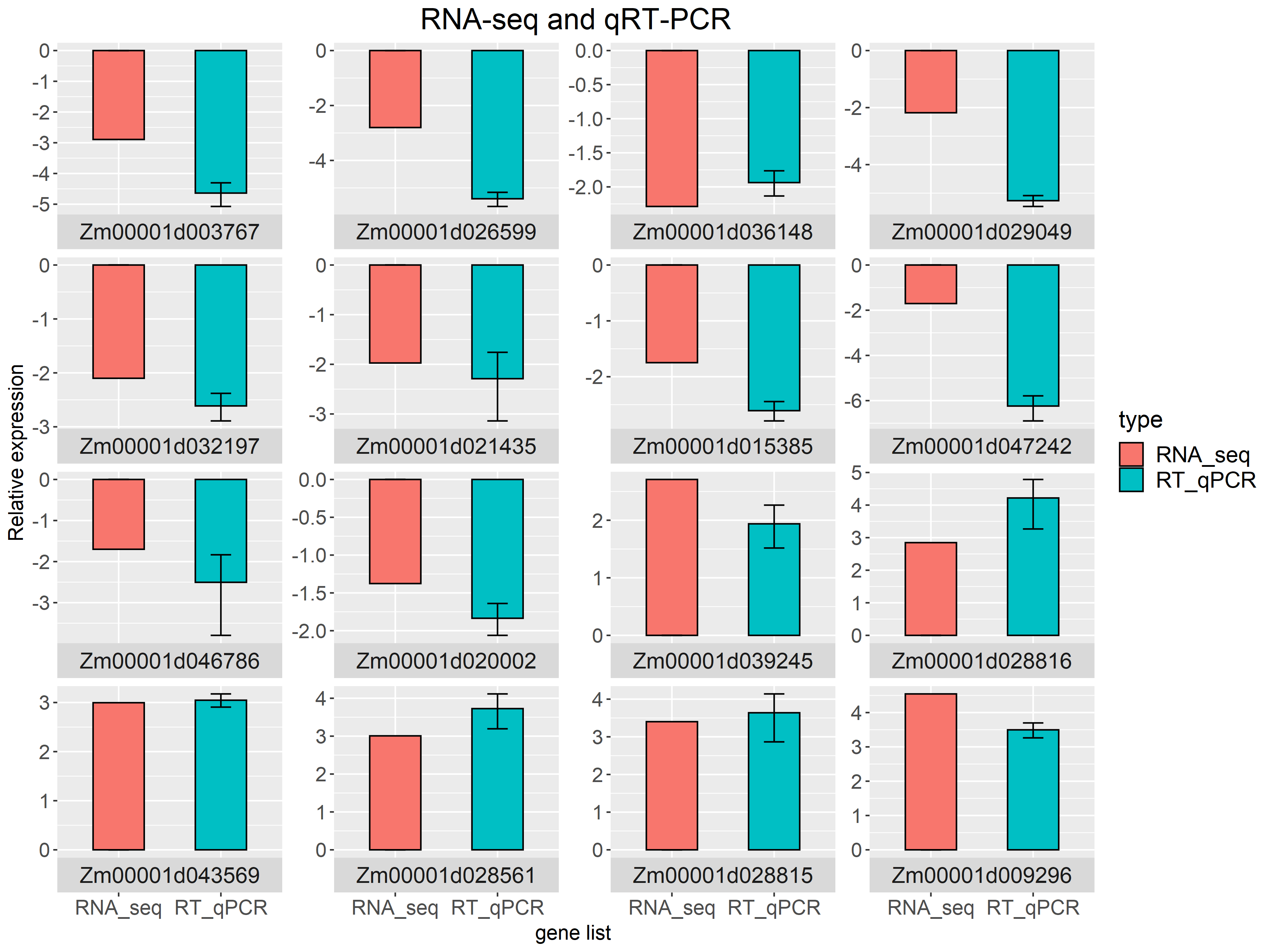

Supplement: Supplementary file 1 [file genes-10-00944-s001.zip › 补充数据/Figure S2.RNA-seq and RT-qPCR result.png]
